# Performance of Polydioxanone-Based Membrane in Association with 3D-Printed Bioceramic Scaffolds in Bone Regeneration

**DOI:** 10.3390/polym15010031

**Published:** 2022-12-21

**Authors:** Letícia Pitol-Palin, Paula Buzo Frigério, Juliana Moura, Livia Pilatti, Letícia Marques Jordão de Oliveira, Elaine Yoshiko Matsubara, Samy Tunchel, Jamil Awad Shibli, Alberto Blay, Sybele Saska, Roberta Okamoto

**Affiliations:** 1 Department of Diagnosis and Surgery, Araçatuba Dental School, São Paulo State University—UNESP, Araçatuba 16015-050, Brazil; 2 M3 Health Indústria e Comércio de Produtos Médicos, Odontológicos e Correlatos S.A., Jundiaí 13212-213, Brazil; 3 Department of Periodontology, Dental Research Division, Guarulhos University, Guarulhos 07009-043, Brazil; 4 Department of Basic Sciences, Araçatuba Dental School, São Paulo State University—UNESP, Araçatuba 16066-840, Brazil

**Keywords:** polydioxanone, 3D-printed scaffolds, β-tricalcium phosphate, hydroxyapatite, bone regeneration, guided tissue regeneration, additive manufacture, synthetic polymer

## Abstract

This study evaluated the bioactivity of 3D-printed β-tricalcium phosphate (β-TCP) scaffolds or hydroxyapatite (HA) scaffolds associated with polydioxanone (PDO) membrane (Plenum^®^ Guide) for guided bone regeneration in rats. Fifty-four rats were divided into three groups (n = 18 animals): autogenous bone + PDO membrane (Auto/PG); 3D-printed β-TCP + PDO membrane (TCP/PG); and 3D-printed HA + PDO membrane (HA/PG). A surgical defect in the parietal bone was made and filled with the respective scaffolds and PDO membrane. The animals were euthanized 7, 30, and 60 days after the surgical procedure for micro-CT, histomorphometric, and immunolabeling analyses. Micro-CT showed an increase in trabecular thickness and a decrease in trabecular separation, even with similar bone volume percentages between TCP/PG and HA/PG vs. Auto/PG. Histometric analysis showed increased bone formation at 30 days in the groups compared to 7 days postoperatively. Immunolabeling analysis showed an increase in proteins related to bone formation at 30 days, and both groups showed a similar immunolabeling pattern. This study concludes that 3D-printed scaffolds associated with PDO membrane (Plenum^®^ Guide) present similar results to autogenous bone for bone regeneration.

## 1. Introduction

Developing technologies and synthetic materials that improve guided bone regeneration (GBR) in the maxillofacial region has a vital role in dentistry. The therapeutic protocol for using GBR includes using membranes as a mechanical barrier associated or not with bone grafts [1,2]. These membranes can be resorbable or non-resorbable, based on a natural or synthetic composition, and bone grafts [1,2]. Among the many advantages of absorbable–synthetic polymers are the reduction of immunogenicity and the creation of an extracellular matrix similar to the host site that favors cell migration, adhesion, proliferation, and differentiation [3,4,5,6,7,8].

Membrane manufacturing with synthetic polymers showed high mechanical performance, biocompatibility, low inflammatory response, and complete degradation [2,9]. Polydioxanone (PDO) is a synthetic polymer consisting of multiple repeating ether–ester units, and is completely colorless, crystalline, and metabolized by the organism [2,10,11]. Its application for creating membranes and scaffolds shows a conductive action for bone regeneration. Plenum^®^ Guide is a synthetic PDO-based membrane widely used for GBR, especially for bone defects [2,10]. The use of PDO membranes promotes a mechanical barrier in the surgical bed, enabling the migration of cells from the surrounding bone tissue and bone formation and reducing the risk of postoperative complications in the long term [1,2,10,11]. In a previous study [2], Plenum^®^ Guide was shown to be an excellent scaffold for the culture of human ASCs, with promising results in regenerating critical bone defects and tissue engineering.

Bioactive ceramics from alloplastic material are entirely synthesized from nonorganic sources [12], including β-tricalcium phosphate (β-TCP) and hydroxyapatite (HA) ceramics [13]. β-TCP has been an attractive alternative for bone substitutes due to its excellent bioactive, incorporating, osteoconductive and resorbable capacity when used in bone grafting [14,15,16,17]. The scientific interest in β-TCP use for alveolar/maxillofacial regions is based on its formulation that enables the creation of scaffolds, which liberate calcium ions when reabsorbed [16]. The macroporosity of β-TCP allows for better vascularization associated with important osteoconductivity. However, it also results in lower mechanical strength of the material under compression, which could limit its more comprehensive application [13,18,19]. HA has a chemical structure very similar to the inorganic bone component, allowing its use as a bioceramic [13,20]. An essential characteristic of synthetic HA is its delayed resorption rate compared to β-TCP due to its high Ca/P and crystallinity of its composition [13,20,21]. This fact gives HA a high mechanical resistance and more significant slowness in its bone replacement process [15,16]. HA nanoparticles promote the adhesion, proliferation, and differentiation of osteoprogenitor cells and are considered bioceramic with potent osteoconductive action [13,20,21,22,23]. 

The decision to use these materials depends on the procedure since the particle size and porosity of these materials influence resorption rates, together with other physical properties that affect the osteoconduction of these bioceramics [8,12,13,18]. 3D printing manufacturing layer by layer through the computer-aided system (CAD and CAM) allows for the creation of custom scaffolds specific in size, roughness, and porosity [24]. Additive manufacturing is considered a subset of rapid prototyping, encompassing techniques to manufacture models and prototypes [24,25,26,27,28,29]. Among its benefits, 3D printing allows more precise control over the dosage distribution of a bio-printed material, reducing waste and promoting individual customization for each type of treatment [27,28]. 3D printing using calcium phosphates has been highlighted for bone regeneration because it enables the production of scaffolds with topography and surface chemical composition similar or closer to the characteristics of bone tissue, thus mimicking an extracellular environment that favors cell–material interactions [29,30,31,32,33,34,35,36]. In addition, 3D printing enables a future association of drugs with biomaterials, promoting increased performance against guided bone regeneration [27,28,37]. Therefore, this study evaluated the bioactivity of 3D-printed β-tricalcium phosphate (β-TCP) scaffolds or 3D-printed hydroxyapatite scaffolds associated with polydioxanone (PDO) membranes (Plenum^®^ Guide) for guided bone regeneration in critical defects in rats calvaria.

## 2. Materials and Methods

### 2.1. PDO Membranes

The PDO absorbable-synthetic membranes are commercially denominated Plenum^®^ Guide (M3 Health Ind. Com. de Prod. Med. Odont. e Correlatos S.A., Jundiaí, SP, Brazil).

### 2.2. Printing of Bioceramic Scaffolds

**Figure 1:**
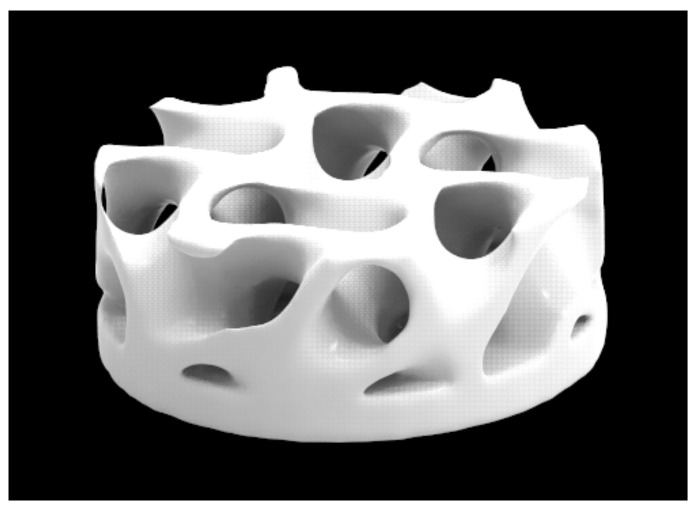
Isometric view of the scaffold STL designed in this work.

STL files of the scaffolds were created using the lattice design software NX Implicit Modeling (version 1953, Siemens Industry Software Inc, USA), applying a gyroid cellular structure (2.4 mm cell size) and resulting in a wall thickness of 0.2 mm (Figure 1). The scaffolds with a 5 mm diameter were 3D-printed using Lithography-based Ceramics Manufacturing (LCM) technology (Cerafab7500, Lithoz GmBH, Wien, Dallas, Austria) with a layer thickness of 25 μm. The feedstock form was a slurry-based polymer mixture with ceramic loading, namely LithaBone TCP 300 (Lithoz GmBH, Wien, Austria) for β-TCP scaffolds and LithaBone HA 400 (Lithoz GmBH, Wien, Austria) for HA scaffolds. The ceramic powder in both slurries had a purity of ≥ 95%, satisfying the criteria for ISO 13175-3:2012 [38]. Parts were manually removed from the build stage and cleaned with an ultrasonic bath, immersed in LithaSol 20 (Lithoz GmBH, Wien, Austria), a cleaning medium for ceramic green parts, and posteriorly with pressurized air (6 bar approx.) to remove any uncured slurry. The green body samples underwent a drying (up to 205 °C), debinding (up to 600 °C), and sintering cycle (hold temperature of 1200 °C for β-TCP and 1300 °C for HA), which lasted 100 h in a muffle furnace with a catalytic converter. The compressive strength of the sintered scaffolds was measured under monotonic uniaxial loading at ambient temperatures at a crosshead speed of 0.5 mm min^−1^ using a universal testing machine (Shimadzu, Kyoto, Japan) according to ASTM C1421-15 [39]. Five cylindrical specimens of each material were tested, resulting in 25.89 ± 3.71 MPa for β-TCP and 4.17 ± 1.18 MPa for HA.

### 2.3. Animals

The study was approved by the Research Ethics Committee of Araçatuba Dental School (#0128-2021), following the Animal Research N3CR guidelines for Reporting In Vivo Experiments (ARRIVE) guidelines [40]. Fifty-four rats (*Rattus norvegicus albinus*, Wistar), male, with 300 g weight and 3 months old, were divided into three groups (*n* = 18): autogenous bone + PDO membrane (Auto/PG); 3D-printed β-TCP + PDO membrane (TCP/PG); and 3D-printed Hydroxyapatite + PDO membrane (HA/PG). The animals were kept in cages in a stable temperature environment (22 °C ± 2 °C, light control cycle 12 light hours, 12 h dark) and balanced diet (NUVILAB, 1.4% Ca, and 0.8% P + water with libitum). 

The sample size for each group was determined using the power test through the website http://www.openepi.com/SampleSize/SSMean.htm (OpenEpi, Version 3, open-source calculator; accessed on 1 July 2021), based on previous results already published [3,4,5]; the averages used for the calculation were 3.06 and 4.898, and the standard deviations were 0.26 and 0.024, with a significance level of 5% and a power of 95% in a one-tailed hypothesis test. Numbers identified the animals and randomly separated them by Microsoft Office Excel software (Microsoft, Redmond, WA, USA), respecting a 1:1 allocation rate for each group.

### 2.4. Surgical Procedure

Animals were fasted for eight hours before the surgical procedure and sedated by intramuscular infiltration of 5 mg/kg xylazine hydrochloride (Dopaser^®^–Laboratórios Calier do Brazil Ltd., Osasco, SP, Brazil) and 50 mg/kg ketamine hydrochloride (Vetaset^®^–Fort Dodge Animal Health Ltd., Campinas, SP, Brazil). The trichotomy and antisepsis of the calvaria region were performed. For each animal, a U-shaped incision was made in the occipitofrontal direction, and the tissue was detached. A surgical defect in the right parietal bone was made using a 5 mm diameter inner drill bit (Zimmer Biomet Holdings Inc.; Warsaw, IN, USA) coupled with a counter-angle with a 20:1 reduction (Angular Part 3624N 1:4, Head 67RIC 1:4, KaVo^®^, Kaltenbach & Voigt GmbH & Co, Biberach, Baden-Württemberg, Germany), mounted on an electric motor (BLM 600^®^; Driller, São Paulo, SP, Brazil), at a speed of 1000 rpm, under irrigation with isotonic 0.9% sodium chloride solution (Fisiológico^®^, Laboratórios Biosintética Ltd., Ribeirão Preto, SP, Brazil). 

**Figure 2:**
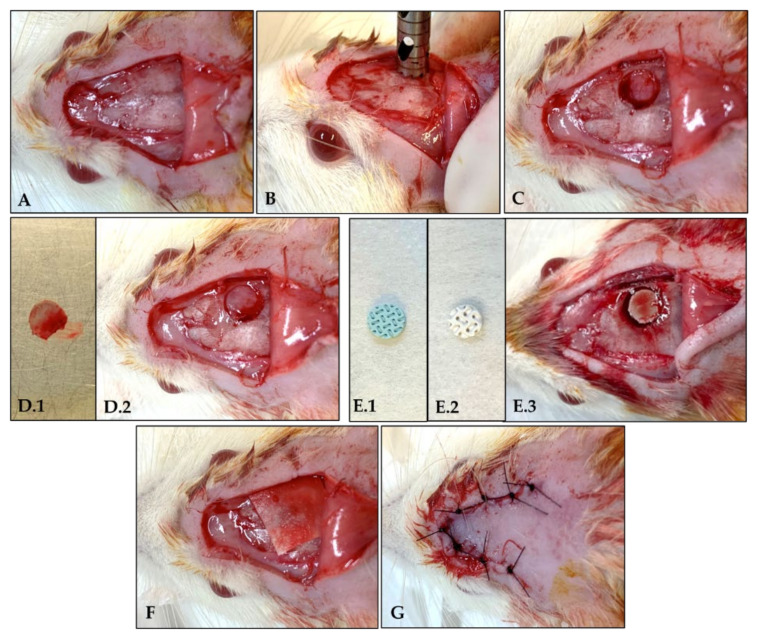
Surgical procedure for creating a critical defect in calvaria. (**A**): U-shaped incision; (**B**): 5 mm diameter inner drill bit positioning for defect creation; (**C**): Osteotomy and parietal bone remotion from inside the defect; (**D.1**): Autogenous bone block; (**D.2**): Placement of autogenous block inside the defect. (**E.1**): 3D-printed HA scaffold; (**E.2**): 3D-printed β-TCP scaffold; (**E.3**): Placement of 3D-printed block inside the defect. (**F**): Placement of customized Plenum^®^ Guide over the defect; (**G**): Tissue closure.

A segment of parietal bone with a 5 mm diameter was removed from inside the defect, maintaining the integrity of the dura mater. The defect was filled with the autogenous bone, 3D-printed β-TCP scaffold (M3 Health Ind. Com. de Prod. Med. Odont. e Correlatos S.A., Jundiaí, SP, Brazil), or 3D-printed HA (M3 Health Ind. Com. de Prod. Med. Odont. e Correlatos S.A., Jundiaí, SP, Brazil) according to the groups. After the application of the biomaterial, the Plenum^®^ Guide with 0.5 × 30 × 40 mm (M3 Health Ind. Com. de Prod. Med. Odont. e Correlatos S.A., Jundiaí, SP, Brazil) was customized and reduced to 12 segments of 10 × 10 mm to be placed over the defect filled with the blocks. The tissue closure was done with monofilament wire (Nylon 5.0, Ethicon, Johnson, São José dos Campos, SP, Brazil) [4] (Figure 1 and Figure 2).

At the immediate postoperative period, each animal received a single intramuscular dose of 0.2 mL of penicillin G benzathine (Pentabiótico Veterinário Pequeno Porte; Fort Dodge Saúde Animal, Ltd., Campinas, SP, Brazil). The animals were euthanized by anesthetic overdosage of xylazine hydrochloride and ketamine hydrochloride at 7 (*n* = 6 per group), 30 (*n* = 6 per group), and 60 days (*n* = 6 per group) after the surgical procedure. Histological analysis was performed on the samples obtained at 7 and 30 days of postoperative; immunolabeling analysis was performed on the samples obtained at 30 days postoperative, and micro-CT analysis was performed on the samples obtained at 60 days postoperative. 

### 2.5. Laboratorial Processing

The laboratory processing of the samples and the proposed analyses were performed in the Laboratory for the Study of Mineralized Tissues, Department of Basic Sciences, Araçatuba Dental School—São Paulo State University (São Paulo Research Foundation—FAPESP—2015/14688-0).

The samples were fixed in 10% formaldehyde solution (Dinâmica^®^ Química Contemporânea Ltd.a., Indaiatuba, SP, Brazil) for 48 h, washed in running water for 24 h, and then decalcified in 10% EDTA (Exodo^®^ Científica Química Fina Industria e Comércio Ltd.a., Sumaré, SP, Brazil) solution for 6 weeks. After decalcification, the calvariae were dehydrated using a sequence of alcohols, diaphanized with xylol, and embedded in paraffin (Labsynth^®^ Produtos-Laboratórios Ltd.a., Diadema, SP, Brazil). Microtomy of the samples was performed to obtain 5 μm-thick slices that were mounted on slides to proceed with the histometric and immunolabeling analyses.

### 2.6. Histological Analysis

For histometric analysis, the slides obtained from the samples 7 and 30 days after surgery were deparaffinized, diaphanized, and rehydrated in a decreasing sequence of alcohols. The sections were stained with hematoxylin (Labsynth^®^ Produtos para Laboratórios Ltd.a., Diadema, SP, Brazil) for 8 min and with 1% eosin (Dinâmica^®^ Química Contemporânea Ltd.a., Indaiatuba, SP, Brazil) for 20 s. The assembled slides were taken to an optical microscope (Leica DMLB, Heerbrugg, St. Gallen, Switzerland) to capture images using a 4×, 10× and, 20× magnification. During image acquisition, the calibrated analyzer performed the histological description in each of the groups following previous studies of the research group. The images (4× magnification) were exported to Adobe Photoshop (Adobe Systems, version CC 2017, San Jose, CA, USA) and were merged to make a panoramic image and perform a descriptive analysis [41].

### 2.7. Immunolabeling Analysis

The immunolabeling process started with deparaffinization and rehydration of the slices, and then endogenous peroxidase activity was inhibited with hydrogen peroxide. Endogenous biotin was blocked with skimmed milk. The primary antibodies (Santa Cruz Biotechnology, Inc., Dallas, TX, USA) were used against Runt-related transcription factor 2—Runx2 (SC101145), osteopontin—OPN (SC10593), osteocalcin–OCN (SC18319), and Tartrate-resistant Acid Phosphatase—TRAP (SC30832). Immunolabeling allows the evaluation of proteins representing different aspects of bone formation and resorption activity. These markers were chosen because they represent osteoblast’s and osteoclast’s activity, considering that Runx2 is a transcription factor that signals the differentiation from pre-osteoblasts to osteoblasts; OPN and OCN label osteoblasts as well as extracellular matrix and the process of mineralization in the initial and late activity respectively, and finally, Trap labels osteoclasts in terms of their resorption activity. 

The analysis was performed with an optical microscope (LeicaR DMLB, Heerbrugg, St. Gallen, Switzerland) by scores means (ordinal qualitative analysis); when the scores were subjected to discrete labeling (+), moderate labeling (++), and intense labeling (+++), it was considered positive for diaminobenzidine (ThermoFisher Scientific, Waltham, MA, USA), taking care to hold negative controls to evaluate the specificity of the antibodies. These scores were established according to previous studies [42,43,44], where light labeling represented about 25% of the immunolabeling area in the blades, moderate labeling represented about 50%, and intense labeling represented about 75%.

### 2.8. Micro-Computed Tomographic (Micro-CT) Analysis

**Figure 3:**
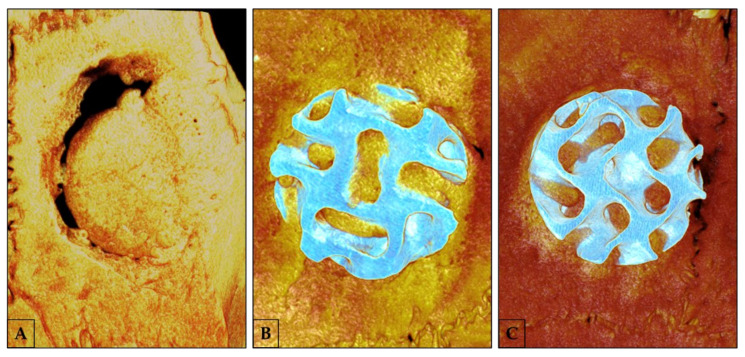
3D images obtained by CTVox software representing the bone formation of Auto/PG, TCP/PG, and HA/PG groups. (**A**): Auto/PG group; (**B**): TCP/PG group; (**C**): HA/PG group.

After euthanasia (60 days), the calvarias were removed, reduced, and fixed in a 10% buffered formalin solution (Dinâmica^®^ Química Contemporânea Ltd.a., Indaiatuba, SP, Brazil) for 48 h and rinsed in running water for 24 h. After fixation, tissue was left in 70% alcohol (Labsynth^®^ Produtos-Laboratórios Ltd.a., Diadema, SP, Brazil) to perform micro-CT. The samples were scanned by a SkyScan 1272 microtomograph (SkyScan 1272 BrukerMicroCT, Leuven, Belgium) using 12 μm-thick sections (90 Kv and 111 μA) using a filter of Al 0.5 mm and a rotation step of 0.6 mm, a resolution of 2016 × 1344 μm, and an acquisition time of 47 min. The images obtained by the projection of X-rays through the samples were stored and reconstituted; the area of interest was determined by the software NRecon (SkyScan, Leuven, Belgium, 2011; Version 1.6.6.0) using built-in filters for a smoothing of 2, a correction of the ring artifact of 5, and a correction of beam hardening of 45%. In the Data Viewer software (SkyScan, Leuven, Belgium, Version 1.4.4 64-bit), the images were reconstructed following observation in three planes (transversal, longitudinal, and sagittal). The CTAnalyser-CTAn software (2003-11SkyScan, 2012 BrukerMicroCT Version 1.12.4.0) was used to evaluate the 3D extension of the bone defect at calvaria (5 mm diameter), selecting 30 slices to perform the analysis. In an axial view of the defect in the custom processing tool, tasks were defined to be applied for morphometric analysis. Following Bruker’s technical note (MN074-Osteointegration: analysis of bone around a metal implant), the region of interest (ROI) was determined for the same volume found in a 5 mm diameter and 30 slices. A threshold was loaded to evaluate and isolate the bone using grayscale values of 40–255. This final volume of interest (VOI) was assessed by 3D analysis of the bone defect (Figure 3). Thus, we were able to define the percentage of bone volume (BV/TV), bone surface (BV), trabecular thickness (Tb.Th), trabecular number and separation (Tb.N, Tb.Sp), and total porosity (Po.Tot) as defined in the manufacturer’s technical note and elsewhere [45].

### 2.9. Statistical Analysis

For statistical analysis, GraphPad Prism 7.03 (GraphPad Software, La Jolla, USA) was used, and Shapiro–Wilk test was assessed and confirmed the normal distribution of the data. ANOVA one-way, followed by Tukey’s posttest to determine differences among the groups, with a significance level of *p* < 0.05.

## 3. Results

### 3.1. Histological Analysis

#### 3.1.1. Histological Analysis at 7 Days of Bone Repair

**Figure 4:**
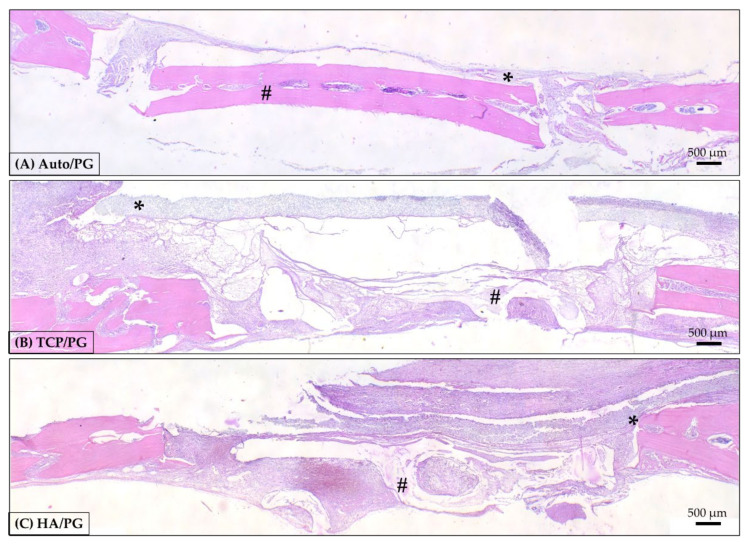
Representative photomicrographs of Auto/PG, TCP/PG, and HA/PG at 7-day histological analysis. (**A**): Auto/PG group; (**B**): TCP/PG group; (**C**): HA/PG group Staining: Hematoxylin and eosin. Legend: * PDO membrane; # autogenous block and 3D-printed blocks. Scale bar: 500 µm (4× magnification).

**Figure 5:**
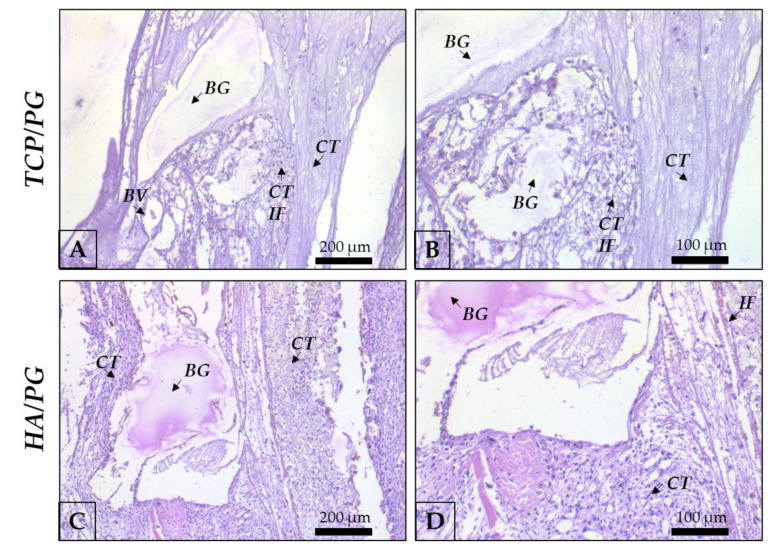
Representative photomicrographs of TCP/PG and HA/PG at 7 days histological analysis. (**A**): TCP/PG 20× magnification; (**B**): TCP/PG 40× magnification; (**C**): HA/PG 20× magnification; (**D**): HA/PG 40× magnification. Staining: Hematoxylin and eosin. Legend: BG: bone graft; CT: connective tissue; BV: blood vessel; IF: inflammatory infiltrate. Scale bar: 200 μm (10× magnification) and, 100 μm (20× magnification).

At 7 days after surgery, in a panoramic view (Figure 4), it was possible to observe the disposition of the blocks, covered by the polydioxanone membrane in all groups. The border of the defects shows connective tissue in the organization. The membrane covers the whole defect, and the blocks remain preserved in the entire structure. In a higher magnification (Figure 5), areas of β-TCP are involved by connective tissue with inflammatory cells, compatible with the repair process step at this chronology. It is important to highlight areas of bone in close apposition of biomaterial remnants. HA block is involved by connective tissue with inflammatory cells, compatible with this step of the repair process. At 7 days, the bone position is close to the biomaterial, as observed. Areas of connective tissue are present close to the biomaterial remnants (Figure 4 and Figure 5). 

#### 3.1.2. Histological Analysis at 30 Days of Bone Repair

**Figure 6:**
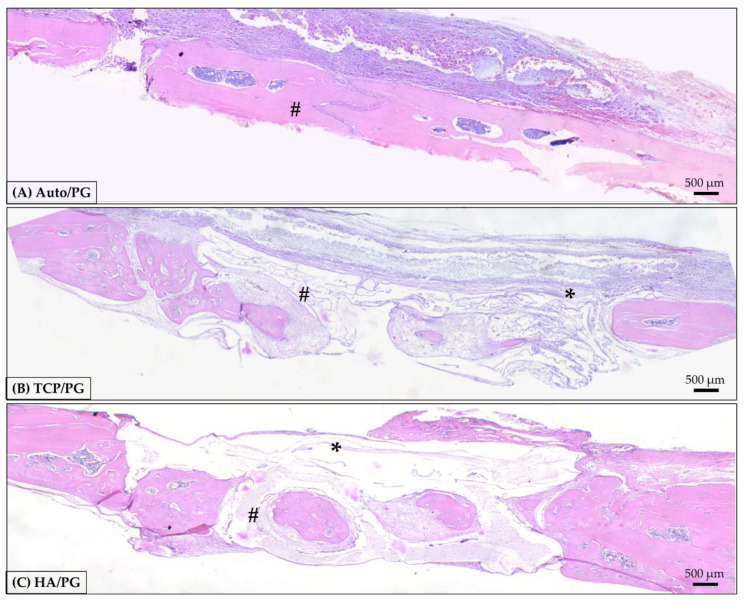
Representative photomicrographs of Auto/PG, TCP/PG, and HA/PG at 30 day histological analysis. (**A**): Auto/PG group; (**B**): TCP/PG group; (**C**): HA/PG group. Staining: Hematoxylin and eosin. Legend: * PDO membrane; # autogenous block and 3D-printed blocks. Scale bar: 500 µm (4× magnification).

**Figure 7:**
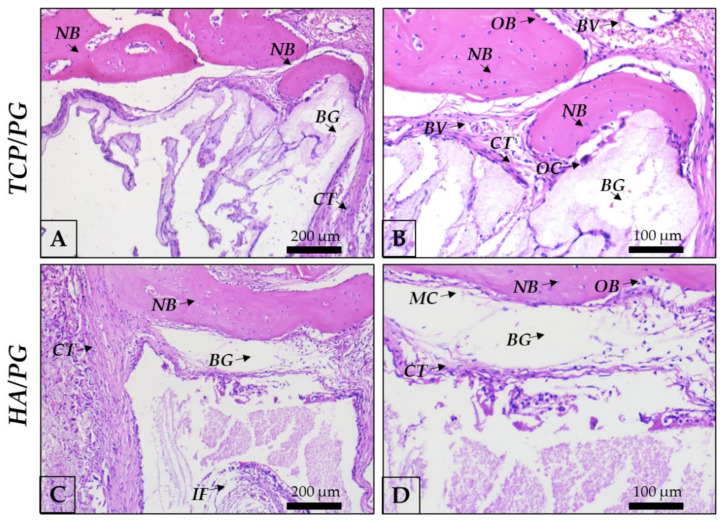
Representative photomicrographs of TCP/PG and HA/PG at 30 day histological analysis. (**A**): TCP/PG 20× magnification; (**B**): TCP/PG 40× magnification; (**C**): HA/PG 20× magnification; (**D**): HA/PG 40× magnification. Staining: Hematoxylin and eosin. Legend: BG: bone graft; CT: connective tissue; BV: blood vessel; IF: inflammatory infiltrate; NB: new bone; MC: mesenchymal cell; OB: osteoblast; OC: osteoclast. Scale bar: 200 μm (10× magnification) and, 100 μm (20× magnification).

At 30 days, in panoramic view (Figure 6), it is possible to observe the evolution of the repair process with bone formed in areas of defect previously filled with the biomaterial blocks. In autogenous grafts, it is important to highlight the closed defect, with the block structure preserved and covered by the membrane. In β-TCP, bone isles were observed, replacing the borders of the block. In the center, incipient bone formation was observed, with connective tissue also filling this area. In HA, there are bone isles in the whole region of the defect, previously filled by the block. Borders show bone formed in the direction of the center of the defect (Figure 6 and Figure 7).

### 3.2. Immunolabeling Analysis

#### Immunolabeling Analysis at 30 Days of Bone Repair

**Figure 8:**
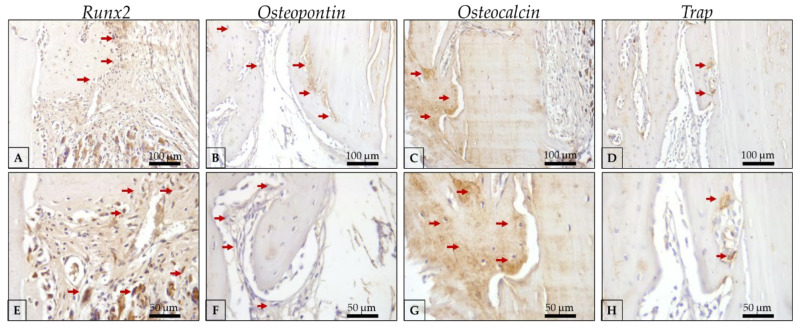
Representative photomicrographs of immunolabeling against Runx2, osteopontin, osteocalcin, and Trap antibodies in the Auto/PG group at 30 days of critical defect repair. (**A**): Runx2 20× magnification; (**B**): OPN 20× magnification; (**C**): OCN 20× magnification; (**D**): Trap 20× magnification; (**E**): Runx2 40× magnification; (**F**): OPN 40× magnification; (**G**): OCN 40× magnification; (**H**): Trap 40× magnification; Counter-staining: Harris Hematoxylin; Red arrows: immunolabeling cells (represent positive labeling for each protein evaluated in the regions of interest). Scale bar: 100 μm (20× magnification) and, 50 μm (40× magnification).

Auto/PG (Figure 8 and Table 1) shows moderate Runx2 labeling (Figure 8a,e) observed in pre-osteoblasts and younger osteoblasts, indicating an important activity of bone repair. OPN labeling (Figure 8b,f) was observed in a moderate expression in osteoblasts and the extracellular matrix, showing the expression of this protein in the repair area. OCN (Figure 8c,g), representing a more mature bone, presents a moderate to intense expression in Auto/PG, showing a mature bone filling the defect. Trap represents osteoclasts undergoing resorption activity that contributes to the substitution of old bone for new bone, consequently activating BMUs. In Auto/PG, at 30 days, we observed a discrete expression (Figure 8d,h).

**Figure 9:**
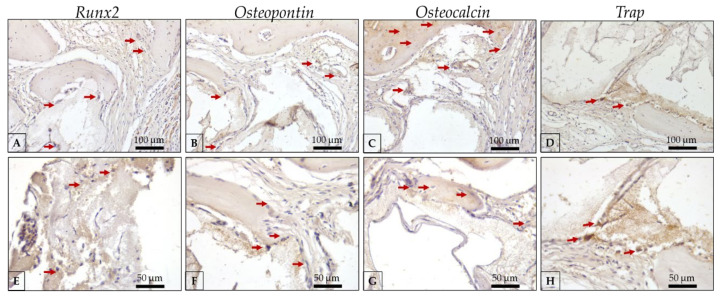
Representative photomicrographs of immunolabeling against Runx2, osteopontin, osteocalcin, and Trap antibodies in the TCP/PG group at 30 days of critical defect repair. (**A**): Runx2 20× magnification; (**B**): OPN 20× magnification; (**C**): OCN 20× magnification; (**D**): Trap 20× magnification; (**E**): Runx2 40× magnification; (**F**): OPN 40× magnification; (**G**): OCN 40× magnification; (**H**): Trap 40× magnification; Counter-staining: Harris Hematoxylin; Red arrows: immunolabeling cells (represent positive labeling for each protein evaluated in the regions of interest). Scale bar: 100 μm (20× magnification) and, 50 μm (40× magnification).

In TCP/PG group (Figure 9 and Table 1), it is important to highlight the discrete immunolabeling of Runx2-positive cells, close to remnants of biomaterial and representing the beginning of osteoblast’s activity in filling the defect (Figure 9a,e). OPN, which labels osteoblasts and the beginning of the biomineralization process, is present in a moderate pattern, with positive labeling for osteoblasts and on the extracellular bone matrix (Figure 9b,f). The maturity of bone formed and number of osteoblasts positive for osteocalcin represents the mineralization of bone formed close to the biomaterial and filling the defect. Moderate labeling for this protein shows an essential activity of this protein that is a marker of the mineralization process (Figure 9c,g). The resorption activity through Trap discrete labeling (Figure 9d,h) is well-represented by the presence of osteoclasts close to the biomaterial and contributing to the renewal of bone.

**Figure 10:**
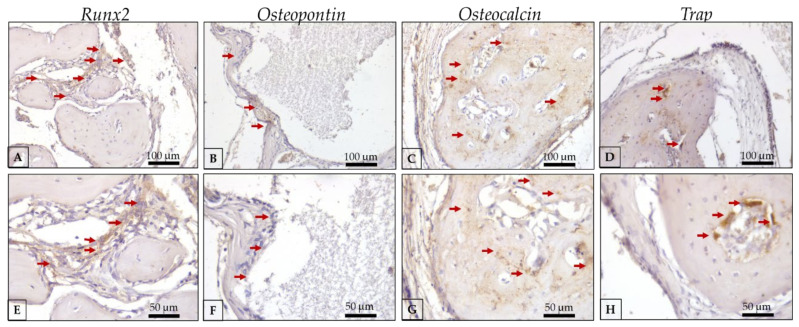
Representative photomicrographs of immunolabeling against Runx2, osteopontin, osteocalcin, and Trap antibodies in the HA/PG group at 30 days of critical defect repair. (**A**): Runx2 20× magnification; (**B**): OPN 20× magnification; (**C**): OCN 20× magnification; (**D**): Trap 20× magnification; (**E**): Runx2 40× magnification; (**F**): OPN 40× magnification; (**G**): OCN 40× magnification; (**H**): Trap 40× magnification; Counter-staining: Harris Hematoxylin; Red arrows: immunolabeling cells (represent positive labeling for each protein evaluated in the regions of interest). Scale bar: 100 μm (20× magnification) and, 50 μm (40× magnification).

In the HA/PG group (Figure 10 and Table 1), we observed the moderate labeling of Runx2, showing an important activity of pre-osteoblasts (Figure 10a,e). OPN is present in a discrete pattern, with osteoblasts with positive labeling of this protein in the area close to the biomaterial (Figure 10b,f). OCN is labeled in a moderate pattern with precipitation onto collagen matrix and showing the maturity of bone formed into the defect and close to the biomaterial (Figure 10c,g). The resorption activity of osteoclasts with discrete labeling of Trap is present in the defect area, representing the renewal of bone and the activation of BMUs that is important for maintaining bone quality (Figure 10d,h).

### 3.3. Micro-Computed Tomographic (Micro-CT) Analysis

#### 3.3.1. Percentage of Bone Volume (BV/TV)

There was no statistically significant difference between the groups (*p* > 0.05). However, the TCP/PG and HA/PG groups showed a higher bone volume percentage than Auto/PG (Table 2).

#### 3.3.2. Trabecular Thickness (Tb.Th)

There was a statistically significant difference when comparing TCP/PG and HA/PG vs. Auto/PG, where the groups with 3D-printed blocks showed a greater thickness of trabeculae (Table 2).

#### 3.3.3. Trabecular Number (Tb.N)

There was a statistically significant difference in the comparison of TCP/PG vs. Auto/PG, where the groups with 3D-printed blocks (TCP and HA) had a lower number of trabeculae per mm^3^ (Table 2).

#### 3.3.4. Trabecular Separation (Tb.Sp)

There was a statistically significant difference in TCP/PG vs. Auto/PG, where the groups with 3D-printed blocks (TCP and HA) had reduced separation between their trabeculae (Table 2).

#### 3.3.5. Total Porosity (Po.Tot)

There was a statistically significant difference when comparing TCP/PG vs. Auto/PG, where the groups with 3D-printed blocks showed a diminution of porosity (Table 2).

### 3.4. Clinical Data

At 7 30 and 60 days of the bone regeneration process, during euthanasia, the animals were necropsied to collect clinical data regarding the healing process. The following aspects were taken into consideration: (1) infection; (2) 3D-printed block out of position; (3) PDO-based membrane out of position; (4) 3D-printed block with non-integration to the bone tissue; and (5) the suture in position.

In both groups, there were no animal losses throughout the experiment and no significant discrepancies between the groups for sample integrity. The Auto/PG group and the TCP/PG and HA/PG groups had rare, compromised samples. The few samples without integrity were excluded to avoid interference in the results obtained in each group.

## 4. Discussion

The search for bone substitutes and membranes is a widely investigated issue in dentistry for treating bone defects that impair the rehabilitation of patients with dental implants and for treating and reconstructing maxillofacial defects [8]. Research using scaffolds, cell-based therapies, membrane technology, and 3D-printing has been used to accelerate the rehabilitative process for patients [16,24,37,46]. Thus, the main hypothesis of this study was that the use of Plenum^®^ Guide membranes could promote guided bone regeneration in different types of bone grafts (autogenous or alloplastic).

Product engineering is a key factor in improving performance and accelerating the repair process [47]. Previous studies [2] have shown that PDO membranes’ morphological structure and surface topography are similar to the extracellular matrix [48], facilitating cell adhesion due to their physicochemical properties enabling biological fluids’propagation. This is also valid for the use of bone substitutes. With the development of technologies, manufacturing products through 3D-printing technology has made it possible to produce structures of complex shapes to aid tissue engineering [25,26,49]. In this study, the association of technologies for developing synthetic materials showed promising results for GBR using Plenum^®^ Guide as a mechanical barrier.

The bone repair processes have well-defined stages in the literature [50], beginning with the recruitment of inflammatory cells and secretion of pro-inflammatory proteins (interleukins and tumor necrosis factor-alpha), which in turn promote the increased synthesis of extracellular matrix, stimulating angiogenesis. The histological analysis showed, at 7 days of repair, the arrangement of the blocks, covered by the polydioxanone membrane in all groups. Due to the architecture of the 3D-printed bioceramics, the center of the defect showed clots, inflammatory cells, and areas of differentiating connective tissue, especially in contact with the remnants of β-TCP and HA. In Auto/PG, these features were not observed in the center of the defect by the presence of the autogenous bone block. At the border of the defects, the presence of organized connective tissue characterizes the chronology of the repair process, where the edges of the defect are faster in forming bone tissue. In this initial period, both materials showed a similar result regarding the tissue repair process. Histological analysis showed, at 30 days, the evolution of the repair process with bone formed in different areas of the defect, previously filled with the biomaterial blocks. Bone islands were observed, replacing the blocks of β-TCP and HA. In the autogenous graft, it is important to highlight the closed defect, with the block structure preserved and partially covered by the membrane. 

Bone regeneration occurs through remodeling commanded by basic multicellular units (BMU) via osteoblasts and osteoclasts to renew bone and mainten of bone quality in the repair process [51]. The immunolabeling analysis enables the observation of tissue responses by expressing of proteins related to the bone repair process [42,43,44]. Runx2 immunolabeling was moderate in Auto/PG, discrete in TCP/PG, and intense in HA/PG, signaling that the group with HA showed an increase in differentiation of pre-osteoblasts to osteoblasts in activity, representing younger cells from the beginning of bone formation compared to the other groups. OPN is a marker of the initial point of biomineralization [42,43,44], showing active osteoblasts and cement lines (reversal lines), indicating activation of the repair process. The HA/PG group showed a discrete labeling of OPN compared to the other groups, corroborating with the increased expression of Runx2 previously observed, indicating a short delay at the beginning of the bone repair process. In the final stages of the mineralization process, OCN precipitation shows osteoblast activity and the biomineralization process with precipitation of this protein with Ca^+^ in the collagen matrix [42,43,44]. Both groups showed moderate OCN expression at 30 days, indicating that the process of Ca^+^ deposition in the matrix occurred similarly in the presence of autogenous bone or synthetic bone substitutes. As a counterpoint, a Trap protein that labels osteoclasts in resorption activity [42,43,44] was seen with a discrete expression in all groups. Thus, the results of the immunolabeling analysis indicate that the repair process, marked by BMUs, occurred similarly when Plenum^®^ Guide was used regardless of the type of graft used.

The integration of bone tissue into the synthetic graft and polymer-based membranes is a factor that indicates the success of the GBR process, since the biocompatibility and biodegradation of the materials indicate the presence of a natural substrate on which cells can attach, proliferate, and differentiate [2,52]. Thus, at 60 days of repair, microtomographic analysis shows the result of the guided bone regeneration process—both groups with synthetic graft present parameters of bone volume percentage that are very similar to those with autogenous grafts. Trabecular parameters are qualitative markers of the bone tissue formed in the defect [53]; thus, using 3D-printed bioceramics promoted a thicker trabecular bone formation than the Auto/PG group. In addition, the TCP/PG group showed decreased separation and increased trabeculae in the bone defect. Visually, in the images provided by CTVox, it is possible to observe the integration of the graft blocks to the bone tissue using the Plenum^®^ Guide. It is worth noting that the architecture of the synthetic blocks aided the bone formation in the center of the defect since their characteristics resemble the bone trabeculae.

Autogenous bone grafting is considered the gold standard in bone regeneration research due to its complete histocompatibility and the presence of all the properties necessary to provide tissue support in GBR [54,55]. However, its use is often not in demand due to its limited availability in some operative sites [54,55]. As new technologies and materials have emerged, the industry’s use of synthetically formulated products has increased significantly [49]. Even though 3D-printing presents several favorable properties, the creation of scaffolds cannot promote all the necessary biological responses. However, in this study, the results obtained in TCP/PG and HA/PG were very similar to the Auto/PG group, indicating that the presence of the PDO-based membrane promoted new bone formation in surgically created defects. These results corroborate another study [2] where Plenum^®^ Guide was compared with Bio-Gide^®^ (Geistlich Pharma AG, Wolhusen, Switzerland), and the groups where collagen-based membranes were used showed a decreased bone formation in surgically created defects.

In vitro results [2] showed that synthetic polymers fabricating PDO membranes effectively increased the migration and growth of human adipose-derived stem cells (hASCs) more than collagen membranes. hASCs are a subgroup of mesenchymal stem cells (MSCs) that exhibit potent regenerative effects by their ability to differentiate into adipogenic, osteogenic, and chondrogenic cells [56,57]. This fact is directly related to the presence of differentiating osteoblast cells shown by the immunolabeling results.

Synergistic action between Plenum^®^ Guide and 3D-printed synthetic blocks must be considered. Several studies show that using biomaterials is less favorable for GBR than autogenous bone grafting [10,58,59]. Thus, 3D-printing synthetic materials based on organic components (such as β-TCP and HA) has proven very successful. Synthetic HA has a delayed resorption rate promoting a limited effect on the quality and quantity of new bone formed after its use in combination with a polymer for alveolar ridge preservation [60]. The results of this present study are promising for the GBR process, associating 3D-printed HA with PDO membranes. However, considering all outcomes, there was a tendency for a discretely improved performance between the association of β-TCP and the PDO-based membrane. This can be explained by the higher porosity and degradation rate of β-TCP compared to other synthetic materials. β-TCP allows for better vascularization, promoting fibrovascular growth and adhesion of osteogenic cells and good resorption compared to bovine bone grafts [10]. These characteristics would allow a faster repair process in GBR.

Studies involving technology in enhancing biomaterials and membranes with cell-based therapies for guided bone regeneration are yet to be performed. A practical approach that combines ASCs with scaffolds allowing the use of different materials (drugs, mRNAs, peptides, growth factors) mimicking natural processes by reproducing an ideal environment for the proliferation and regeneration of injured tissue is the future of tissue engineering. Cell therapies have been studied in various areas, from the treatment of degenerative diseases to their applicability in dentistry, which has shown positive results for the treatment of periodontal diseases, endodontic treatments, and guided bone regeneration. This is a preliminary study, but the results were promising and indicate new research goals, with new scaffolds and even different technologies and innovations that aim to optimize bone repair.

## 5. Conclusions

Polydioxanone-based membranes (Plenum^®^ Guide) promoted guided bone regeneration independent of the type of graft used. Furthermore, using of 3D-printed bioceramics scaffolds promoted the bone tissue neoformation with similar characteristics to the autograft bone and, in some respects, improved of the quality of repaired bone. However, these findings support a new era of synthetic biomaterials, membranes, and bone graft substitutes, to be able promote predicable clinical performance.

## Figures and Tables

**Table 1:** Immunolabeling scores in Auto/PG, TCP/PG, and HA/PG at 30 days of bone repair. Antibodies against Runx2, OPN, OCN, and Trap.

	Auto/PG	TCP/PG	HA/PG
Runx2	++	+	+++
OPN	++	++	+
OCN	++	++	++
Trap	+	+	+

Scores are evaluated at the border/center of the defect. Discrete labeling (+), moderate labeling (++), and intense labeling (+++).

**Table 2:** Micro-CT results: Auto/PG, TCP/PG and HA/PG ± standard deviation (SD).

	Auto/PG	TCP/PG	HA/PG	Statistical Difference
BV/TV (%)	82.710 ± 6.757	87.950 ± 1.732	84.360 ± 6.323	no
Tb.Th (mm)	0.2504 ± 0.027	0.3318 ± 0.008	0.3144 ± 0.049	Auto/PG vs. TCP/PG = 0.0027 Auto/PG vs. HA/PG = 0.0189
Tb.N (1/mm)	2.945 ± 0.184	2.533 ± 0.247	2.700 ± 0.167	Auto/PG vs. TCP/PG = 0.0351
Tb.Sp (mm)	0.226 ± 0.043	0.170 ± 0.021	0.182 ± 0.020	Auto/PG vs. TCP/PG = 0.0442
Po.Tot (%)	23.960 ± 7.294	12.050 ± 1.732	16.120 ± 6.452	Auto/PG vs. TCP/PG = 0.0214

Mean values, standard deviations, and statistically significant differences were presented by Auto/PG, TCP/PG, and HA/PG groups at 60 days of bone repair.

## Data Availability

The data presented in this study are available on request from the corresponding author.
